# Mo_2_CT_*x*_ MXene-based non-enzymatic electrochemical sensor for selective detection of hydrogen peroxide in colorectal cancer cells

**DOI:** 10.1039/d6na00025h

**Published:** 2026-05-19

**Authors:** Shruthi Venkataraman, Vasanth Magesh, Chandramohan Govindasamy, Raji Atchudan, Dhanraj Ganapathy, Sandeep Arya, Ashok K. Sundramoorthy

**Affiliations:** a Centre for Nano-Biosensors, Department of Prosthodontics and Materials Science, Saveetha Dental College and Hospitals, Saveetha Institute of Medical and Technical Sciences India ashok.sundramoorthy@gmail.com; b Department of Community Health Sciences, College of Applied Medical Sciences, King Saud University P. O. Box 10219 Riyadh 11433 Saudi Arabia; c School of Chemical Engineering, Yeungnam University Gyeongsan 38541 Republic of Korea; d Department of Physics, University of Jammu Jammu Jammu and Kashmir 180006 India

## Abstract

Accurate and real-time monitoring of hydrogen peroxide (H_2_O_2_) in biological environments is critical for understanding redox-regulated processes associated with cancer progression. In this study, we developed, for the first time, a novel non-enzymatic electrochemical sensor based on two-dimensional molybdenum carbide MXene (Mo_2_CT_*x*_) for the selective detection of H_2_O_2_, with particular importance in human cancer cell applications. The synthesized Mo_2_CT_*x*_ MXene was broadly characterized to confirm its layered morphology, surface terminations, and structural integrity. When coated onto a glassy carbon electrode (GCE), the MXene-modified glassy carbon electrode (MX-GCE) exhibited excellent electrocatalytic activity toward H_2_O_2_ reduction at a low operating potential of −0.45 V. The MX-GCE demonstrated a linear response range from 0.49 to 78.3 µM with a low detection limit of 1.02 µM and a rapid response time of ∼2 s. Interference analysis indicated that the MX-GCE exhibited high selectivity for H_2_O_2_ in the presence of common biological interferents. The practical applicability of the sensor was validated through real sample analysis by monitoring of extracellular H_2_O_2_ generated by cancer cells upon ascorbic acid induced stimulation. These results established that the Mo_2_CT_*x*_ MXene modified electrode could be used as a stable and effective sensing platform for H_2_O_2_ detection in complex biological environments. This new sensor will be useful for oxidative stress monitoring and cancer diagnostics.

## Abstract 2

Mo_2_CT_*x*_ MXene-modified electrode exhibited high electrocatalytic activity and was employed for the non-enzymatic detection of H_2_O_2_ in SW480 colorectal cancer cells, providing a rapid, stable and sensitive platform for monitoring of oxidative stress.

## Graphical abstract

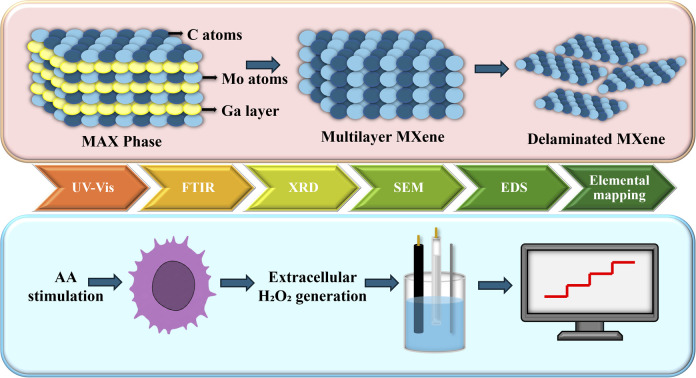

## 1. Introduction

MXenes are a family of two-dimensional (2D) transition-metal carbides, nitrides, and carbonitrides synthesized by the selective removal of the “A” layer from MAX phases (M_*n*+1_AX_*n*_). Their general formula is M_*n*+1_X_*n*_T_*x*_, where “M” denotes an early transition metal (Ti, Mo, V, Sc, Nb, or Cr), “*n*” denotes the number of layers of carbon or nitrogen signified as “X”, and T_*x*_ signifies the surface terminations (–F, –O, –OH).^1^ These MXenes are highly ordered and possess unique “accordion” layer-like morphology, where the M–X bonds are strongly covalent, which gives the material high conductivity, structural rigidity, and chemical stability.^2^ Introduction of surface terminations during the etching processes increases the hydrophilicity, redox activity, and catalytic properties of MXenes.^3^ These features differentiate MXenes from other 2D materials, such as graphene or transition-metal dichalcogenides, providing them with their unique properties.^4^ Due to several advantages, MXenes are suitable for a wide range of applications in energy storage, electronics, and biomedical applications.^5,6^

Among the various MXene compositions reported to date, Ti-based MXenes have been the most extensively investigated, largely due to their biocompatibility^7^ and electrochemical properties.^8^ Moreover, their synthesis procedures are well studied, allowing reproducible production of high-quality sheets in both multilayer and delaminated forms. Consequently, a substantial portion of MXene research focused on characterisation techniques, and MXene applications using Ti-based systems.^9,10^ Though Ti_3_C_2_T_*x*_ sheets are highly conductive, they are prone to rapid oxidation, which interferes with their long-term stability.^11^ This has directed attention to the need for exploring alternative compositions, such as Mo-based MXenes, which offer distinct catalytic properties and emerging advantages for specialised applications.^12^ For the first time, this work potentially explores the electrochemical properties of Mo_2_CT_*x*_ nanosheets as an amperometric probe for the reduction of hydrogen peroxide (H_2_O_2_).

Colorectal cancer ranks as the third most commonly diagnosed cancer worldwide, contributing to about 10% of all cancer cases, and is the second leading cause of cancer-related deaths globally, as reported by the World Health Organization (WHO) in 2023.^13^ In 2022, it is estimated that more than 1.9 million new cases of colorectal cancer and more than 900 000 deaths occur worldwide every year.^13^ Geographical variations in incidence and mortality rates have been observed, with the highest incidence rates in Europe, Australia, and New Zealand.^13^ The risk of developing colorectal cancer increases with age, and the majority of cases are observed in individuals over 50 years old. Predisposing conditions, such as genetic conditions, family history, and unhealthy lifestyle choices, could increase the risk.^15^ In the early stages, the disease often presents with no significant clinical manifestations, emphasizing the need for regular screening to diagnose the cancer and begin treatment.^15^ It is also reported that the incidence rates of colorectal cancer have been declining in high-income countries due to the implementation of effective screening programmes. Prognosis strongly depends on the stage of diagnosis, with early-stage diagnosis associated with better survival outcomes compared to advanced stages.^14^

Reactive oxygen species (ROS) play an essential role in maintaining cellular homeostasis, regulating proliferation, differentiation, and programmed cell death.^16^ Within the ROS family, H_2_O_2_ occupies a central position due to its relatively long life span,^17^ membrane permeability,^18^ and the ability to function as a signalling messenger.^19^ Under physiological conditions, intracellular H_2_O_2_ levels are regulated by antioxidant systems; however, in cancer cells, this balance is significantly disrupted.^20^ Elevated H_2_O_2_ production in these cells is linked to changes such as oncogene activation, mitochondrial stress, and faster metabolic activity. Together, these factors facilitate the growth of cancer cells rapidly, causing damage to the DNA, angiogenesis, and increasing the metastatic potential.^21^ Accurately quantifying H_2_O_2_ in the tumour microenvironment is therefore critical not only for understanding redox-driven cancer progression but also for evaluating the biochemical impact of therapeutic agents that modulate oxidative stress.^22^

Conventional methods for H_2_O_2_ detection are available such as fluorescent probes,^23^ colorimetric assays,^24^ chemiluminescence,^25^ and enzyme-linked systems.^26^ Even though they offer high sensitivity in *in vitro* setups, they face major limitations in biological systems. Fluorescent dyes lack selectivity and are prone to photobleaching,^27^ while enzyme-linked systems mostly depend on the stability of peroxidases that tend to degrade rapidly in complex cellular environments.^28^ These approaches also require complex instruments, multistep sample preparations, and may provide false positive results.^29^ These drawbacks limit their use for real-time, *in situ*, and quantitative monitoring of H_2_O_2_ in living cells, while advancements in electrochemical sensing platforms offer high selectivity, rapid response, miniaturisation, and compatibility with microenvironments.^30,31^

Electrochemical sensors, especially non-enzymatic platforms, are growing as powerful alternatives, governed by electrocatalytic properties and characteristics of the electrode materials.^31,32^ Within the MXene class, Mo_2_CT_*x*_ have emerged as unique catalysts for sensing applications. Delaminated MXenes expose a high density of catalytically active Mo sites and surface terminations capable of enhancing the adsorption and reduction of H_2_O_2_.^33^ Another crucial advantage of Mo_2_CT_*x*_ is their hydrophilicity, due to the presence of surface functional groups, promoting stable dispersion in aqueous media, thereby increasing biosensing efficiency.^11^ The nature of MXenes also allows stable dispersion in aqueous media, which is crucial for detecting sub-micromolar concentrations of H_2_O_2_ released from cells.^34^ Their negatively charged surface also helps in minimising non-specific interference from other intracellular species, thereby improving selectivity. Incorporating Mo_2_CT_*x*_ MXene in electrochemical sensing not only addresses stability and performance concerns but also opens up opportunities for real-time monitoring of oxidative signalling in cancer cells, providing insights into tumour biology and enabling improved diagnostic and therapeutic strategies.

**Fig. 1:**
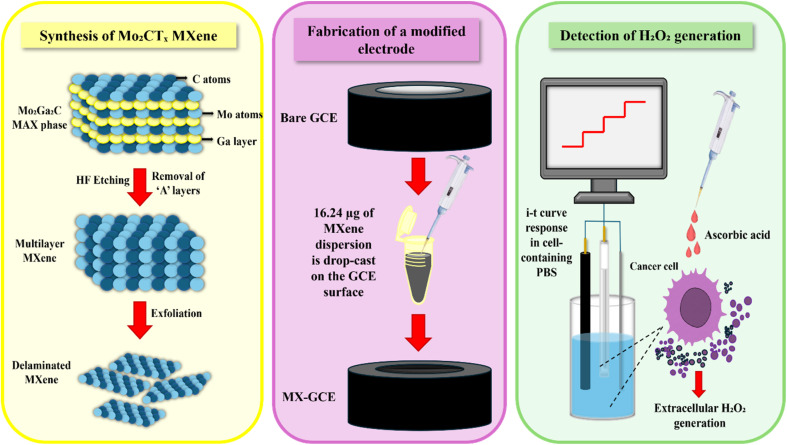
Schematic illustration of the overall experimental workflow for the fabrication of an H_2_O_2_ sensor using the MX-modified GCE.

In this study, for the first time, we demonstrated a novel electrochemical sensor based on Mo_2_CT_*x*_ MXene for the selective detection of H_2_O_2_ released by SW480 colorectal cancer cells. The as-synthesised MXene was thoroughly characterised to visualise its morphology and verify its elemental distribution. The experimental parameters have been optimised (effect of pH, catalyst loading, and stability of the modified electrode) for the sensitive and selective detection of H_2_O_2_ in live cells. Amperometric detection of H_2_O_2_ is also demonstrated for real-time monitoring of the ROS in cells using the Mo_2_CT_*x*_ modified electrode. Overall, this study highlights the previously unexplored potential of Mo_2_CT_*x*_ MXene as an electrochemical sensing material and provides a practical platform for real-time monitoring of oxidative signalling in cancer cells, which (eqn (1)–(3)) depicts the electron reduction pathway of H_2_O_2_.^35^Fig. 1 shows the schematic workflow employed for the detection of H_2_O_2_ using a Mo_2_CT_*x*_ MXene-modified electrode (MX-GCE).

1 H_2_O_2_ + H^+^ + e^−^ → H_2_O + ˙OH

2 ˙OH + H^+^ + e^−^ → H_2_O

3 H_2_O_2_ + 2H^+^ + 2e^−^ → 2H_2_O

## 2. Experimental

### 2.1. Chemicals and instruments

Molybdenum carbide (∼325 mesh, 99.5%) was acquired from Sigma-Aldrich, India. The gallium ingot (Ga, 99.9999%, trace metal basis) was obtained from Acros Organics, Thermo Fisher Scientific. Hydrochloric acid (HCl, 35%) was obtained from Sigma-Aldrich, India. Hydrofluoric acid (HF, 48%) was purchased from NICE Chemicals, India. Dopamine hydrochloride, l-ascorbic acid, d-glucose, and sodium chloride were purchased from SRL Pvt. Ltd, India, and uric acid was purchased from NICE Chemicals, India. All the electrochemical studies were conducted in 0.1 M phosphate buffer solution (PBS, pH 7.4). The purchased chemicals were used as received, with no additional purification. All solutions were prepared using deionised water with a resistivity of 18.2 MΩ cm.

### 2.2. Synthesis of Mo_2_CT_*x*_ MXenes

The Mo_2_Ga_2_C MAX phase was prepared using molybdenum carbide (MoC) powder and gallium metal ingot. These were mechanically ground together in a molar ratio of 1 : 1 using a mortar and pestle. Upon grinding, the MoC–Ga mixture transformed into a homogeneous silver-gray paste. The paste was then allowed to rest overnight. This paste was then transferred into a quartz tube and filled with argon gas to establish an inert atmosphere. The mixture was then subjected to thermal annealing at 850 °C for 48 h in a tubular furnace. Followed by annealing, the mixture was left undisturbed to cool naturally at room temperature. The resultant material exhibited a blackish-gray appearance, indicative of successful MAX phase formation. To eliminate the gallium metal and gallium oxide, 1 g of Mo_2_Ga_2_C powder was immersed in 9 M HCl for 24 h. The vigorous evolution of hydrogen gas bubbles during this step confirmed the dissolution of surface Ga and Ga_2_O_3_. The purified powder was subsequently recovered by vacuum filtration, thoroughly rinsed with DI water, and dried at 80 °C under vacuum for 24 h. The final Mo_2_Ga_2_C powder was then subjected to structural and morphological characterization.^30^

The Mo_2_CT_*x*_ MXene was obtained through selective removal of the Ga atomic layers from the Mo_2_Ga_2_C MAX phase *via* a modified chemical etching approach. 0.5 g of Mo_2_Ga_2_C powder was dispersed into 30 mL of a prepared etching solution consisting of 12 M HCl, DI water, and 48% HF in a volumetric ratio of 6 : 1 : 3. The suspension was continuously stirred at 400 ± 20 rpm and maintained at 75 °C ± 5 °C in an oil bath for a total duration of 120 h. The etched product was subsequently washed repeatedly with DI water through centrifugation at 3500 rpm for 5 min per cycle. Before each centrifugation step, 35 mL of fresh DI water was added to the MXene sediment in the centrifuge tube, which was then manually shaken for approximately 1 min to ensure thorough dispersion. This washing procedure was continued until the pH of the collected supernatant exceeded 6. Upon reaching the target pH, one additional DI water wash was carried out to finalize the purification.

To obtain delaminated few-layer or monolayer MXene (d-Mo_2_CT_*x*_), the washed precipitate was redispersed in 30 mL of N_2_-degassed DI water. A 1 mL aliquot of this dispersion was further diluted with 5 mL of DI water (1 : 5, v/v ratio) and subjected to bath sonication for 5 min at a controlled temperature of 22–25 °C. The sonicated dispersion was then allowed to stand undisturbed at room temperature for 20 min. The stable, colloidal few-layer MXene suspension formed at the top was carefully collected and used for subsequent physicochemical characterization and electrochemical measurements.^30^

### 2.3. Preparation of the Mo_2_CT_*x*_ modified GCE

A GCE with a 3 mm diameter was carefully polished with 0.05 µm alumina slurry in a polishing cloth to achieve a highly reflective, mirror-like surface. After that, the GCE was rinsed and dried in a vacuum oven at 50 °C. On the GCE surface, 7 µL of a well-dispersed MXene suspension (2.3 mg mL^−1^) was drop-cast and dried for 30 minutes. After drying, the modified MX-GCE was stabilised in 0.1 M PBS by potential sweeping between −0.7 V and +0.1 V at a scan rate of 50 mV s^−1^ for 10 cycles.

### 2.4. Characterisation

To observe the UV-visible absorption spectrum of the MXene, a UV-Jasco spectrophotometer was used. A Fourier Transform Infrared (FTIR) spectrophotometer (ALPHA II, Bruker, Germany) was used to successfully determine the various functional groups present in the material. The crystallinity of the material was confirmed by obtaining an X-ray diffraction (XRD) pattern with the use of an X-ray diffractometer (D8-Advance XRD BRUKER, Billerica, MA, USA). The XRD pattern was obtained with Cu Kα radiation of wavelength 1.5406 Å. The surface morphology of the MXenes was investigated with a field emission scanning electron microscope (SEM) (Carl Zeiss, USA). An energy-dispersive X-ray (EDS) (Bruker, Germany) spectrum was obtained along with elemental mapping to further confirm the surface characteristics.

### 2.5. Electrochemical measurements

All electrochemical analyses were carried out using an electrochemical workstation, CHI 760E (CH Instruments, Austin, TX, USA). This study used a three-electrode system, with Ag/AgCl maintained in 1 M KCl as the reference electrode, a GCE as the working electrode, and a platinum wire used as the counter electrode to perform all the experiments. Cyclic voltametric analyses were carried out between −0.7 V and 0.1 V, whereas amperometric responses were recorded at a fixed potential of −0.45 V.

### 2.6. Cell culture preparation for real sample analysis

The human colorectal adenocarcinoma cell line SW480 was obtained from the National Centre for Cell Science (NCCS), Pune, and maintained under standard cell culture conditions. Cells were cultured in high-glucose Dulbecco's Modified Eagle Medium (DMEM) supplemented with 10% fetal bovine serum (FBS) and 1% penicillin–streptomycin, and incubated at 37 °C in a humidified atmosphere containing 5% CO_2_. SW480 cells exhibited an epithelial-like, adherent morphology and were routinely grown as a monolayer, with medium replacement performed every 2–3 days. Cells were subcultured at 60–80% confluence by washing with sterile phosphate-buffered saline (PBS), followed by a brief trypsin–EDTA treatment to detach the cells. The enzymatic action was neutralized using a complete culture medium, and the resulting cell suspension was gently resuspended to ensure uniformity before reseeding. Cell viability and density were determined using a trypan blue exclusion assay with a hemocytometer and approximately 2.6 × 10^4^ viable cells were seeded in 5 mL of DMEM for subsequent experiments. For the selective detection of extracellular H_2_O_2_ release, ascorbic acid (AA) (0.1 M) was used as a stimulant. 100 µL of the AA was carefully added to the electrochemical cell to record the current response.^36^ On the other hand, to the same concentration of cells, a known concentration of H_2_O_2_ was spiked to further investigate the sensor's ability to detect spiked H_2_O_2_.

## 3. Results and discussion

### 3.1. UV-vis spectroscopy

**Fig. 2:**
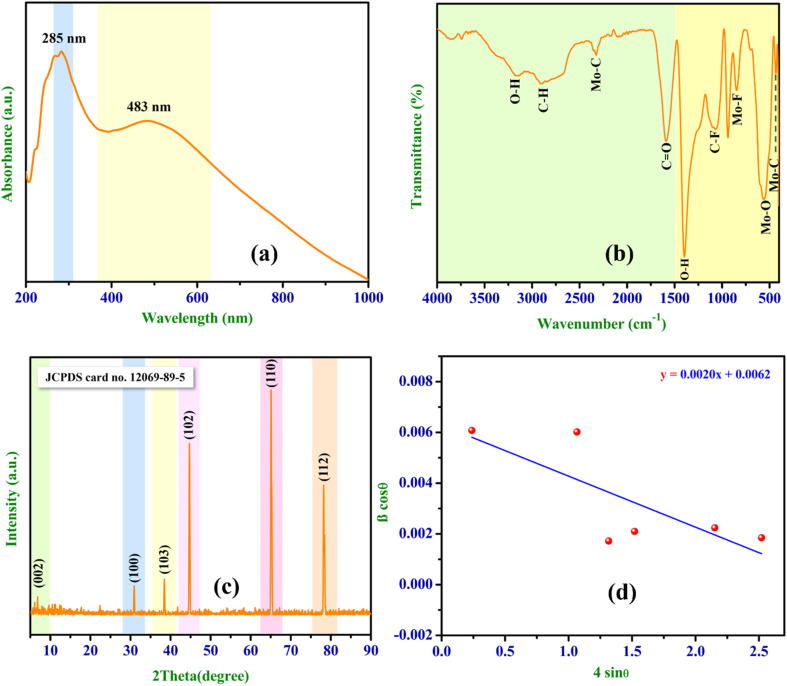
(a) UV-vis spectrum, (b) FTIR spectrum, (c) XRD data and (d) Williamson–Hall plot of Mo_2_CT_*x*_ MXene.

The absorption spectrum of Mo_2_CT_*x*_ is shown in Fig. 2a, which demonstrated a distinct absorption peak at 283 nm. This peak position is likely attributed to the n → π* transitions, indicating the presence of lone pairs of electrons on oxygen and other surface groups.^11,37^ The absorption shoulder bands at 219 nm and 245 nm indicated the influence of surface terminations such as –OH, –F, or =O groups.^38^ Another peak at 264 nm is attributed to π → π* transitions within the Mo–C framework.^39^ The presence of a broad hump around 483 nm is due to d–d transitions of Mo atoms^34^ responsible for the electronic conductivity of the material. A long tail in the NIR region with decreasing absorbance extending from 600 nm indicated that the material becomes transparent with lower energy electronic transitions. Therefore, the UV-vis absorption spectrum successfully validated the optical properties of the Mo_2_CT_*x*_ as reported elsewhere.^40^

### 3.2. FTIR analysis

FTIR spectroscopy was employed to examine the surface chemistry and the functional terminations in relation to Mo_2_CT_*x*_ MXene. The obtained spectrum reveals distinct peaks in the functional group region (4000–1500 cm^−1^) and characteristic vibrational modes in the fingerprint regions (1500–400 cm^−1^). As illustrated in Fig. 2b, the broad peak observed between 3600 and 3000 cm^−1^ corresponds to O–H stretching vibrations, indicating the presence of hydroxyl surface terminations introduced during the etching process, while the O–H bending mode found at 1396 cm^−1^ further validated the confirmation.^41^ The presence of aliphatic hydrocarbons is reflected with stretching vibrations at 2906 cm^−1^, arising from trace residues. The FTIR spectrum also revealed the presence of C=O stretching at 1589 cm^−1^, which denoted the presence of carbonyl groups.^37^ A broad peak at 1069 cm^−1^ corresponds to C–F stretching vibration due to the introduction of fluorine-containing surface terminations.^42^ A pronounced peak at 938 cm^−1^ and 562 cm^−1^ denoted the stretching and bending vibrations, revealing the surface oxidation of molybdenum species.^43^ Other characteristic peaks in the fingerprint region, corresponding to Mo–C stretching vibrations (434 cm^−1^), confirmed the carbide framework retention from the MAX phase. Another peak found at 2325 cm^−1^ further supported the presence of Mo–C stretching vibrations.^44^

### 3.3. XRD analysis

The XRD pattern of the as-prepared Mo_2_CT_*x*_ is presented in Fig. 2c. The values of interlayer spacing were calculated using Bragg's law (eqn (4) and (5)) with respect to the corresponding 2*θ* values.^45^

4 *nλ* = 2*d* sin *θ*

5 *d* = *nλ*/2 sin *θ*

The low-angle diffraction peak at 6.82° corresponds to the (002) plane of Mo_2_CT_*x*_, which denoted the increased interlayer *d*-spacing (12.95 Å) arising from the successful removal of the Ga-layer from the precursor.^46^ However, the peak shift towards lower angles compared to previously reported studies indicated a large increase in the interlayer *d*-spacing within the framework.^47,48^ This distinct shift confirmed the effective exfoliation and interlayer expansion, which is the characteristic of delaminated MXenes. Despite the interlayer expansion, the carbon and molybdenum atoms maintain their hexagonal lattice within each sheet. However, the diffraction angles found at 30.92° (100), 44.70° (101), and 65.08° (110) revealed the loss of three-dimensional structure between the sheets, which distinguishes delaminated MXene from the MAX phase.^47^ To further support it, comparative XRD analysis was performed with the MAX phase precursor (Fig. S1). The XRD pattern of the pure Mo_2_Ga_2_C MAX phase exhibited diffraction peaks at 9.8° (002), 34.3° (100), 37.51° (103), 39.9° (008), 42.6° (105), 53.7° (108), and 61.3° (110).^37^ The shift observed from 9.8° in the MAX phase to 6.82° in the MXenes supported the increased interlayer *d*-spacing in the MXenes.^49,50^ The peak at 39.97° in the MAX phase, relative to the peak at 38.7°, is substantially reduced in intensity, indicating the removal of Ga layers. However, the complete disappearance of this peak is not seen. This might be due to the trace levels of Ga present in the Mo_2_CT_*x*_ MXene.^37^ While the low-angle reflections confirmed the MXene formation, the high-angle reflections indicated the partial retention of Mo–C ordering, depicting scattering from planes, further confirming the Mo–C stacking.

The average crystallite size was calculated using the Scherrer equation^51^ (eqn (6)) and the Williamson–Hall (W–H) plot^52^ (eqn (7)), and the obtained values were estimated as 55 nm and 22 nm, respectively.^53^

6 *D* = *kλ*/(*β* cos *θ*)

7 *β* cos *θ* = (*kλ*/*D*) + 4*ε* sin *θ*

Eqn (7) corresponds to the Uniform Deformation Model (UDM), which assumes that the lattice strain is uniformly distributed along all crystallographic directions. In this model, *k* denotes the dimensionless shape factor (0.9), *λ* represents the wavelength of the Cu Kα radiation, *D* is the average crystallite size, *θ* is the Bragg angle, *β* refers to the full width at half maximum (FWHM) of the diffraction peak, and *ε* corresponds to the lattice strain.^53^ The reduction in crystallite size obtained from the W–H plot (Fig. 2d) can be attributed to instrumental limitations as well as lattice strain. The slope of the W–H plot corresponds to a value of 0.002, which indicated that the contribution of lattice strain to peak broadening is negligible.^53^ Hence, this observation reveals that the observed peak broadening is predominantly governed by crystallite size, with minimal influence from lattice strain.^54^ These results were comparable with previously established studies and validated the MXene formation.^55,56^

### 3.4. FESEM analyses of Mo_2_CT_*x*_

**Fig. 3:**
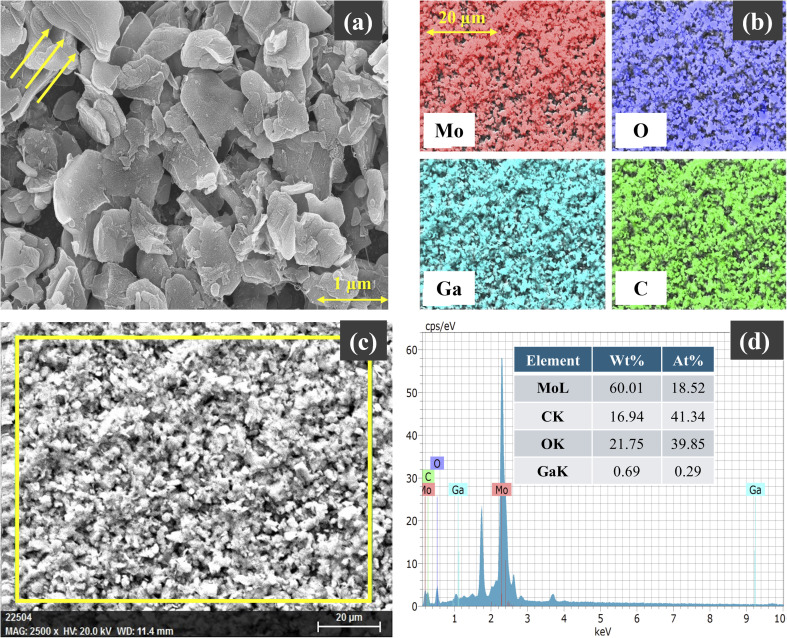
(a) FESEM image of the as-synthesised Mo_2_CT_*x*_ MXene, showing characteristic layered morphology (yellow arrows); scale bar: 1 µm. (b) EDS elemental mapping indicating the uniform distribution of Mo, C, and O elements, scale bar: 20 µm. (c) A low magnification FESEM image depicting the large surface area morphology and homogeneous aggregation of Mo_2_CT_*x*_ MXene; scale bar: 20 µm. (d) EDS spectrum with corresponding elemental composition of Mo_2_CT_*x*_ MXene (wt% and at%).

The SEM image reveals a characteristic accordion-like morphology of MXenes (Fig. 3a, yellow arrows). The stacked and partially delaminated sheets exhibited sharp edges with interlayer separation and exfoliation, confirming the structural formation of MXene from the parent MAX phases.^57^ Along with the morphology, the elemental compositions were also analysed using elemental mapping (Fig. 3b), which showed a uniform distribution of Mo, C, and O across the scanned region. The presence of oxygen is attributed to the surface terminations (–O, –OH) introduced during the synthesis process.^58^ The scanned area in Fig. 3c indicated that the Mo_2_CT_*x*_ flakes have a rough texture, denoting the absence of bulk particles, which in turn indicates the effective conversion from the MAX phase to 2D MXene structures. The EDS spectrum (Fig. 3d) confirmed the presence of dominant elements with quantitative analysis providing 60.01 wt% Mo, 16.94 wt% C, and 21.75 wt% O. The negligible Ga content (0.69 wt%) further supports the effective etching process. Moreover, the conversion of the bulk MAX phase precursor to an exfoliated MXene structure was also investigated by FESEM analysis (Fig. S2a and b). The MAX phase exhibits a dense, bulk, and irregularly shaped morphology. The EDS spectrum revealed higher wt% (36.99) and at% (18.43) of Ga, which are significantly lower in the case of MXene. However, there are some traces of Ga present in the MXene structure, which were also evident from the XRD analysis. These analyses further confirmed the successful formation of Mo_2_CT_*x*_ MXene.^34,59,60^

## 4. Electrochemical characterisation of the modified electrode

**Fig. 4:**
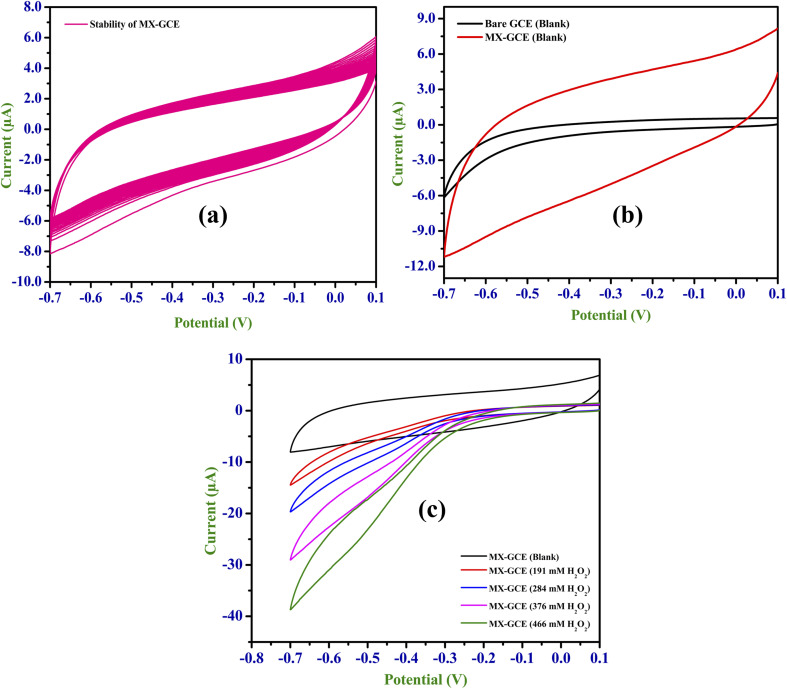
(a) Cyclic voltammograms (CVs) of MX-GCE recorded over 25 cycles in 0.1 M PBS (PBS 7.4) at a scan rate of 50 mV s^−1^, (b) comparative CVs recorded with bare GCE and MX-GCE in 0.1 M PBS (pH = 7.4) at a scan rate of 50 mV s^−1^, and (c) CVs recorded using MX-GCE in the presence of various concentrations of H_2_O_2_ (191, 284, 376, and 466 mM) in comparison with bare GCE in 0.1 M PBS (pH = 7.4) at a scan rate of 50 mV s^−1^.

The stability of the MX-GCE is monitored by potential scanning for 25 cycles in 0.1 M PBS from −0.7 to 0.1 V, as shown in Fig. 4a. The absence of current decrease or distortion confirmed the strong adhesion of the Mo_2_CT_*x*_ film on the GCE surface. The enhanced current response exhibited by the MX-GCE is compared with the current produced by the bare GCE in Fig. 4b, which highlighted the increased electrochemical activity of the modified electrode. The electrochemical sensing performance of the MX-GCE towards H_2_O_2_ is investigated in comparison to bare GCE as shown in Fig. 4c. A significant current difference is observed with the modified electrode in the presence of different concentrations of H_2_O_2_ in 0.1 M PBS (pH 7.4). This data confirmed the role of the MX-GCE in facilitating H_2_O_2_ electro-reduction, thereby improving electrochemical activity compared to the bare GCE. This behaviour is characteristic of a catalyst that increases the rate of electron transfer rather than overpotential reduction. The increased catalytic current at the MX-GCE in the presence of H_2_O_2_ at the same cathodic potential indicated the improved electrocatalytic kinetics, which is a suitable for amperometric sensing.^61^ It is evident that the MX-GCE enhances the rate of H_2_O_2_ reduction rather than shifting the onset potential.^62^ The absence of a reversible redox couple indicated that the Mo_2_CT_*x*_ film did not undergo significant faradaic self-redox processes in the operating potential. The enhanced cathodic peak current in the presence of increasing concentrations of H_2_O_2_ is attributed to the direct electrocatalytic reduction at the Mo active sites.^63^

### 4.1. Electrochemical impedance spectroscopy (EIS) analysis

**Fig. 5:**
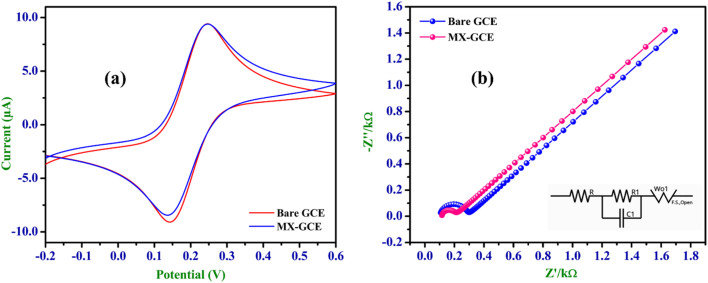
(a) CVs recorded with bare GCE and MX-GCE in 0.1 M KCl with 5 mM [Fe(CN)_6_]^3−/4−^ at a scan rate of 50 mV s^−1^. (b) Nyquist plots obtained from EIS for bare GCE and MX-GCE in 0.1 M KCl containing 5 mM [Fe(CN)_6_]^3−/4−^ in the frequency range of 0.01 Hz to 10 kHz with an amplitude of 5 mV. Inset: The equivalent Randles-type circuit used for fitting the impedance data.

EIS was performed in the presence of 5 mM [Fe(CN)_6_]^3−^/[Fe(CN)_6_]^4−^ to demonstrate the electron-transfer kinetics (Fig. 5a) at the electrode–electrolyte interface. The parameters include the frequency ranging from 0.01 Hz to 10 kHz with an amplitude of 5 mV. Fig. 5b presents the Nyquist plots of the bare and MX-GCE, which were fitted using the equivalent circuit model. The similar intercepts for both the electrodes correspond to solution resistance (*R*_s_), confirming that the experimental conditions are identical. The MX-GCE exhibited a lower charge-transfer resistance (*R*_ct_) compared to the bare GCE, supporting the accelerated electron-transfer, which is attributed to high electrical conductivity. Both electrodes displayed a linear Warburg response (*W*), confirming the diffusion-controlled mass transport (Table 1).

**Table 1:** EIS values obtained from the bare and modified GCEs

Sample	*R*_s_ (Ω)	*R*_ct_ (Ω)	*C*_dl_ (F)	*W* (Ω)
Bare GCE	103	179	5.847 × 10^−7^	1.649 × 10^−3^
MX-GCE	115	86	2.08 × 10^−6^	1.635 × 10^−3^

**Fig. 6:**
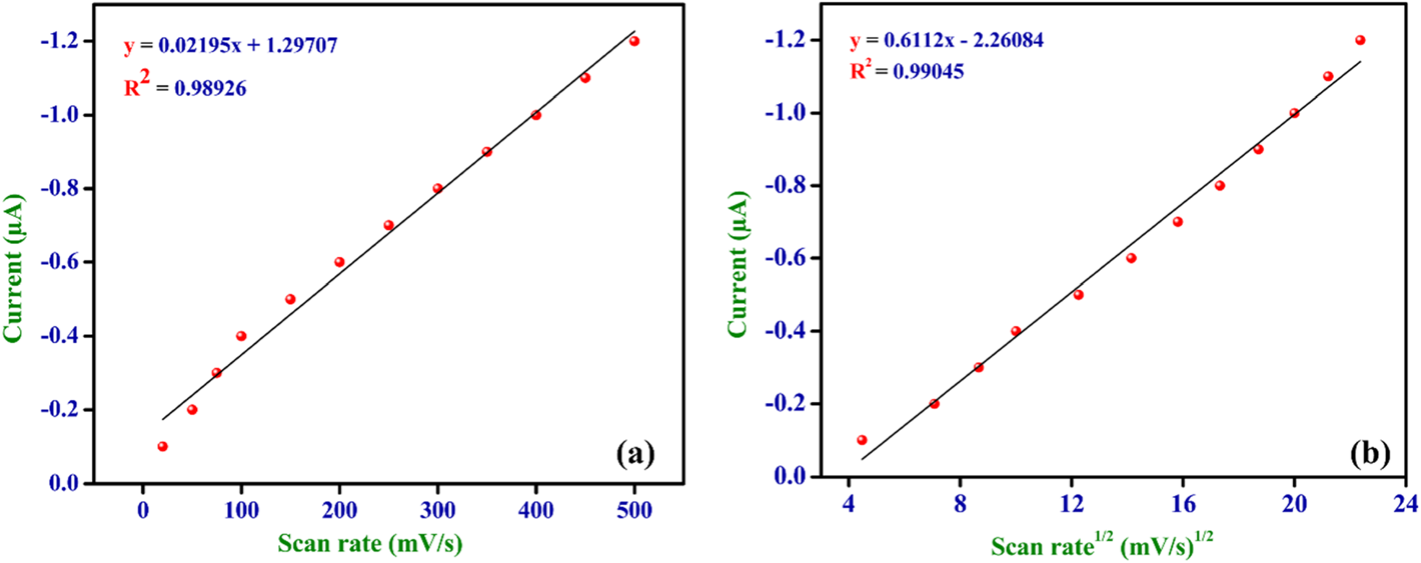
(a) Linear relationship between cathodic peak current and scan rate (20–500 mV s^−1^) recorded in 0.1 M PBS (pH = 7.4) in the presence of 284 mM H_2_O_2_, (b) corresponding linear dependence of cathodic peak current *vs.* the square root of scan rate recorded in 0.1 M PBS (pH = 7.4) containing 284 mM H_2_O_2_ (between the scan rates of 20 and 500 mV s^−1^).

The electrochemically active surface areas (ECSA) of the bare and the modified electrode (Fig. 6a) were calculated using the Randles–Ševčík formula (eqn (8)):^62^

8 *I*_p_ = (2.69 × 10^5^)*n*^3/2^*AD*^1/2^*Cν*^1/2^

where ‘*I*_p_’ is the cathodic reduction peak current, ‘*n*’ is the number of electrons transferred, ‘*D*’ denotes the diffusion coefficient of the redox probe (6.7 × 10^−6^ cm^2^ s^−1^),^64^ ‘*ν*’ denotes the scan rate (0.05 V s^−1^), and ‘*C*’ denotes the concentration (5 × 10^−6^ mol cm^−3^). Finally, the ECSAs of the bare GCE and MX-GCE were calculated as 0.115 cm^2^ and 0.108 cm^2^, respectively. This reveals that the bare GCE has slightly higher ECSA than the MX-GCE. This phenomenon can be explained by the restacking behaviour of 2D Mo_2_CT_*x*_ nanosheets. Although MXenes possess a high theoretical surface area, the planar drop-cast film can undergo face-to-face van der Waals stacking, which partially blocks access to the diffusing redox probe.^65^ Generally, ECSA is calculated using the Randles–Ševčík equation and reflects the area accessible to the outer-sphere redox probe, and it is not equivalent to the catalytically active surface for inner-sphere H_2_O_2_ reduction.^65,66^

### 4.2. Effect of scan rate on H_2_O_2_ reduction

The influence of scan rate on the electrocatalytic response of Mo_2_CT_*x*_ was investigated in the presence of 284 mM H_2_O_2_ at various scan rates (20 mV s^−1^, 50 mV s^−1^, 75 mV s^−1^, and 500 mV s^−1^) (Fig. S3b). The linear plot between the effect of scan rate and the cathodic peak current of the bare GCE is illustrated in Fig. S3a. This clearly revealed that the cathodic peak current exhibited a strong linear dependence (*n* = 3) on the square root of scan rate (*R*^2^ = 0.990) (Fig. 6a), indicating that the electrochemical process is a predominantly diffusion-controlled process in the presence of H_2_O_2_.^67^ On the other hand, a linear relationship between the peak current and the scan rate (*R*^2^ = 0.989) (Fig. 6b) was also recorded, which also denoted a surface-controlled process.^62^ Thus, the relationship between log *i* and log *ν* has been assessed through eqn (9) and (10), where ‘*i*’ denotes current, ‘*b*’ is the slope, and ‘*ν*’ is the scan rate. The plot (Fig. S4) revealed the *b* value to be 0.3 at three different potentials (−0.3 V, −0.45 V, −0.5 V). In general, when the *b* value is found to be ∼0.5, the system follows a diffusion-controlled process.^68^

9 *i* = *aν*^*b*^

10 log *i* = *b* × log *ν* + log *a*

11
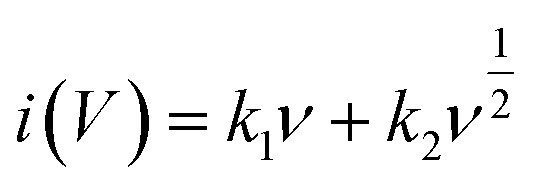
12
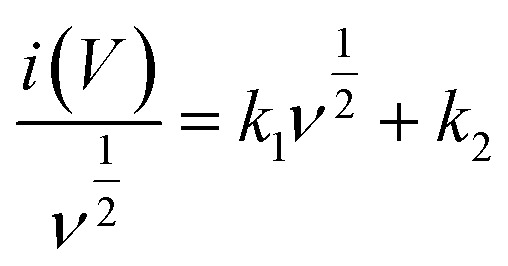


This can be further discussed by quantifying the capacitive contribution, which proved that the electron kinetics are primarily diffusion-controlled. According to Dunn's method, the total charge stored can be divided into a faradaic contribution (diffusion-controlled) and a capacitive contribution.^69^ Hence, from eqn (11) and (12), the % capacitance was calculated to be 13.6%, 26.3%, and 36.7% at scan rates of 50 mV s^−1^, 150 mV s^−1^, and 250 mV s^−1^, respectively, at a potential of −0.45 V. This also further supported the dominance of the diffusion-controlled mechanism of the Mo_2_CT_*x*_ system.

### 4.3. Stability of the modified electrode

The stability of the MX-GCE was investigated by CV in 0.1 M PBS (pH 7.4) at a scan rate of 50 mV s^−1^. For this study, using the MX-GCE, CVs were recorded at different time intervals of 1, 4, and 7 days to determine the inter-day stability of the modified electrode (Fig. S5a). Also, the intraday stability was investigated under the same experimental conditions. Three independent experiments were carried out by CVs on the same day to study the intra-day stability of the modified electrode (Fig. S5b). Inter-day stability of the modified electrode resulted in an RSD of 5.2%, and intraday stability resulted in an RSD value of 2.2%. Therefore, it is evident that the Mo_2_CT_*x*_/GCE possesses excellent stability, which is suitable for electrochemical sensing applications.

## 5. Electrochemical sensing of H_2_O_2_

### 5.1. Effect of pH

The effect of solution pH on the electrocatalytic reduction of H_2_O_2_ at the MX-GCE was examined over a pH range of 2–7. As shown in Fig. S6a, the cathodic peak current response strongly depends on the electrolyte pH, with acidic conditions producing higher reduction currents due to increased proton availability. Fig. S6b reveals the relationship between the cathodic peak current and the effect of pH. At low pH values (pH 2–4), enhanced cathodic currents are observed (Fig. S6), indicating proton-coupled electron transfer during H_2_O_2_ reduction.^70^ Simultaneously, a noticeable shift of the potential towards more positive values occurs with decreasing pH, confirming the direct involvement of protons in the reaction mechanism.^71^ At pH 8, H_2_O_2_ degradation is accelerated compared to acidic conditions, due to the increase in OH^−^ ions, thereby producing a negligible current response.^72^ This proton-coupled electron transfer is explained by comparing the potential with respect to different pH levels in a linear regression plot. This plot provided a slope of −0.050 mV per pH, close to the Nernstian slope value of 59 mV per pH (Fig. S6c).^73^ Also, extremely acidic conditions may compromise the electrode stability and are not suitable for physiologically relevant samples. At neutral pH, the MX-GCE exhibited a stable and well-defined current response. Therefore, pH 7.4 was chosen for subsequent electrochemical sensing, as it closely mimics the biological environment and ensures reliable sensing without hindering the electrocatalytic efficiency.^74^

### 5.2. Effect of catalyst loading

The effect of Mo_2_CT_*x*_ MXene loading onto the electrode surface has been investigated towards H_2_O_2_ by drop casting various concentrations of 3 µL (6.69 µg), 5 µL (11.6 µg), and 7 µL (16.24 µg) of MXene suspension (2.3 mg mL^−1^) in 0.1 M PBS (pH 7.4) at a scan rate of 50 mV s^−1^. As depicted in Fig. S7a, increasing the catalyst concentration led to an enhancement in cathodic current, confirming that a higher amount of Mo_2_CT_*x*_ provides an increased number of accessible active sites and improved electron-transfer pathways for H_2_O_2_ reduction. The CVs further revealed that the electrode modified with 16.2 µg MXene exhibited a more pronounced response than the other concentrations. In Fig. S7b, the bar graph reveals that the average catalytic currents for 6.6 µg and 11.6 µg are slightly higher than that of 16.2 µg. But this is observed with substantially larger error bars with SD = 5.94 × 10^−6^, 7.70 × 10^−6^, and 1.81 × 10^−6^ for 6.6 µg, 11.6 µg, and 16.2 µg, respectively, indicating higher standard deviation and poorer reproducibility. This might be attributed to non-uniform MXene coverage and partial exposure of the GCE surface, which led to inconsistent electron-transfer behaviour.^75^ In contrast, the 16.2 µg-modified electrode exhibited a slightly lower mean current but significantly reduced standard deviation, demonstrating a more homogeneous MXene film, stable interfacial contact, and reproducible electrocatalytic response.^76^ Therefore, 16.2 µg was selected as optimal due to its stability and reproducibility, which are important parameters for electrochemical sensing.

### 5.3. Amperometric detection of H_2_O_2_

**Fig. 7:**
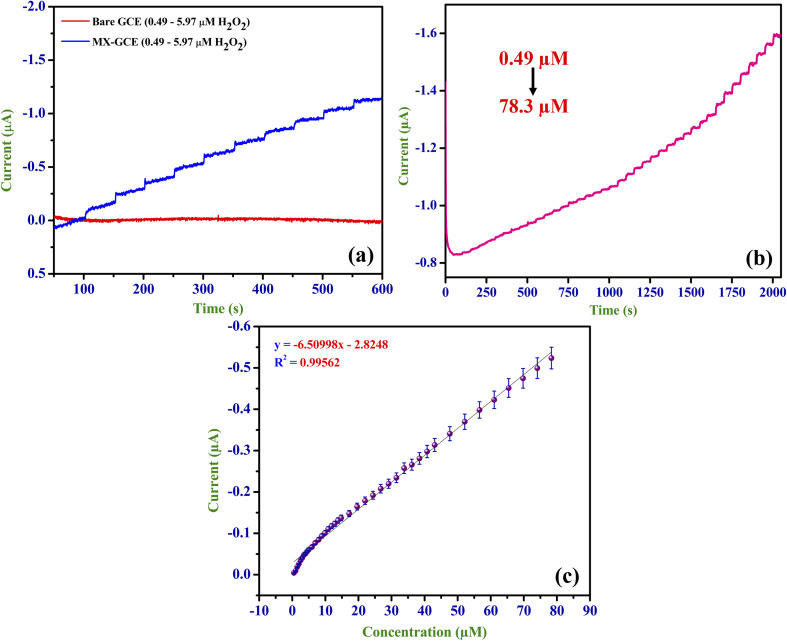
(a) Amperograms recorded with bare GCE and MX-GCE at a fixed potential of −0.45 V in 0.1 M PBS (pH = 7.4) at 720 rpm with stepwise standard addition of H_2_O_2_ between the concentrations of 0.5 and 5.97 µM. (b) Amperograms recorded in MX-GCE at a fixed potential of −0.45 V in 0.1 M PBS (pH 7.4) at 720 rpm with successive additions of H_2_O_2_ in a linear range of 0.49–78.3 µM, (c) a calibration plot obtained from four independent amperometric measurements using MX-GCE in 0.1 M PBS (pH = 7.4) at 720 rpm in the presence of H_2_O_2_ (0.49–78.3 µM).

The amperometric response of the MX-GCE towards H_2_O_2_ was investigated at a fixed potential of −0.45 V in 0.1 M PBS (20 mL) (pH of 7.4). At this potential, the current responses are stable, evident from the CV responses. Also at −0.45 V, the selectivity of H_2_O_2_ is increased, suppressing the interference from other oxidisable species like AA, UA, and DA, which do not undergo electro-reduction at this potential range. The successive additions of H_2_O_2_ produced well-defined and stepwise increases in cathodic current. In contrast, the bare GCE exhibited a significantly lower current response (Fig. 7a), highlighting the role of Mo_2_CT_*x*_ in improving the catalytic current and providing increased active surface area.^77^ As shown in Fig. 7b, the current increases proportionally with the analyte concentration over linear segments, indicating stable electrocatalytic activity at the MXene interface. A linear response range of the sensor was observed from 0.49 µM to 78.3 µM H_2_O_2_ by amperometry. To demonstrate the relationship between the reduction peak current and the concentration of H_2_O_2_, a calibration plot (Fig. 7c) was made from 0.49 µM to 78.3 µM, with *R*^2^ of 0.995, highlighted the excellent linear relationship. Furthermore, the limit of detection (LOD) for H_2_O_2_ was calculated using the following equation (eqn (13)), where SD corresponds to the standard deviation of the blank signals (*n* = 3) and *S* corresponds to the slope from the calibration curve obtained from Fig. 7c:^78^

13 LOD = 3 × SD/*S*

With the help of a calibration plot, the slope (*S*) was calculated to be 6.5099 × 10^−9^ µA µM^−1^. Thus, the LOD value for H_2_O_2_ was estimated to be 1.02 µM. The various analytical parameters, such as linear range, applied potential, and LOD of the present method, are compared with those of some previously reported electrochemical sensors in Table 2. In contrast to previously reported MXene-based sensors that rely on other nanomaterials to enhance current response, this work demonstrated the intrinsic Mo–C framework to achieve low-potential, biologically relevant H_2_O_2_ quantification. On the other hand, previous studies predominantly focused on breast, cervical, or prostate cancer models.^79–81^ Our work is the first to demonstrate direct amperometric detection of H_2_O_2_ from SW480 colorectal cancer cells using a Mo_2_CT_*x*_ system.

**Table 2:** Comparison of H_2_O_2_ detection with previously reported MXene electrodes^a^

Catalyst/composite	Technique used	Applied potential	LOD	Linear range	Sensitivity	Real sample analysis	Ref.
rGO–Ti_3_C_2_–MWCNTs	Amperometry	−0.25 V	0.3 µM	1–60 µM and 60–9.77 mM	235.2 µA mM^−1^ cm^−2^ and 103.8 µA mM^−1^ cm^−2^	MCF-7 and 4T1 cells	82
MXene–Co_3_O_4_	Cyclic voltammetry, linear sweep voltammetry		0.5 µM	up to 75 µM	5.40 ± 0.26 µA mM^−1^	MDA-MB-231 and DU145	83
MXene/CMCS@HRP	Amperometry	0.4 V	0.29 µM	0.5 µM to 3 mM	56.45 µA mM^−1^ cm^−2^	HeLa cells	84
Mn_*x*_O_*y*_/Ti_3_C_2_	Amperometry	+0.8 V	1 nM	0.05–6 µM and 20–650 µM	17 420 µA mM^−1^ cm^−2^	MCF-7 cells	85
Au/MXene/PU	Amperometry	+0.4 V	3.928 nM	0.1–100 µM	0.0127 µA µM^−1^	MDA-MB-231 cells	86
Mo_2_CT_*x*_	Amperometry	−0.45 V	1.02 µM	0.49–78.3 µM	6.50998 × 10^−9^ µA µM^−1^	SW480	This work

a rGO – reduced graphene oxide, Co_3_O_4_ – cobalt oxide, CMCS – compositing biocompatible carboxymethyl cellulose, HRP – horseradish peroxidase, Mn_*x*_O_*y*_ – manganese oxide, PU – polyurethane.

### 5.4. Interference study

**Fig. 8:**
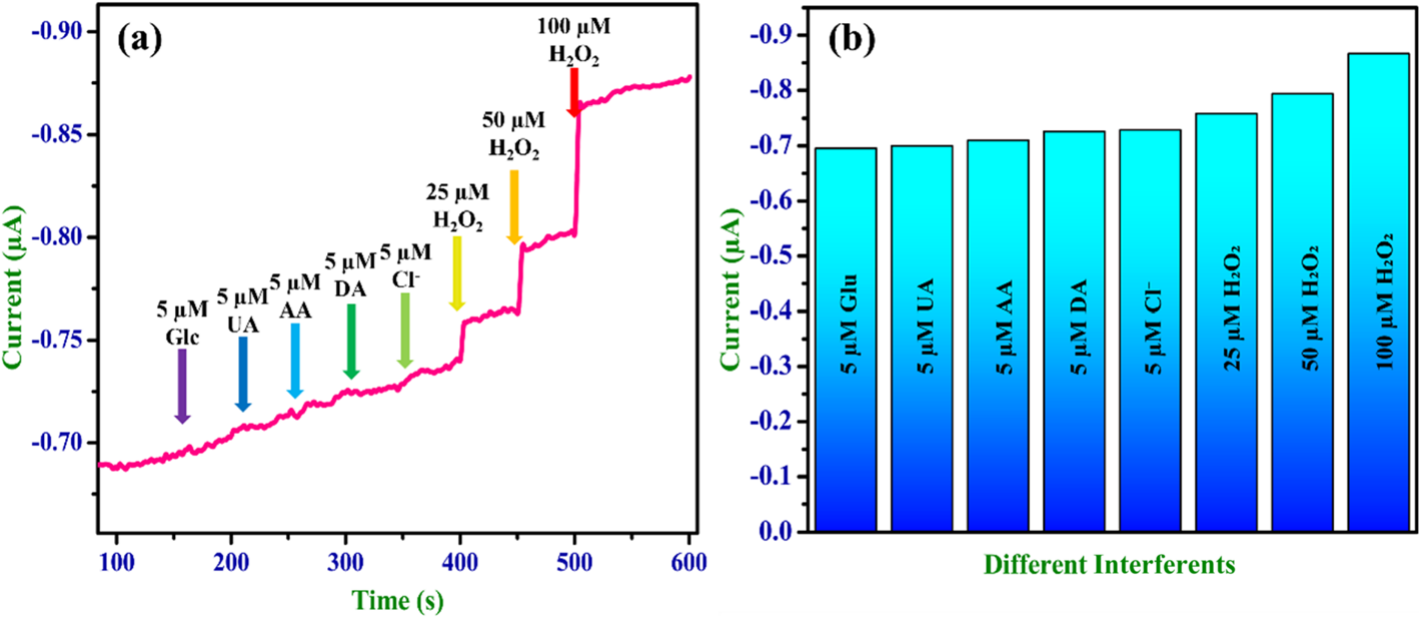
(a) Amperograms recorded using MX-GCE in the presence of H_2_O_2_ (25, 50, and 100 µM) with different interfering species such as Glc, UA, AA, DA, and Cl^−^ (each concentration = 5 µM) in 0.1 M PBS (pH = 7.4) with continuous stirring at 720 rpm. (b) Bar diagram showing the steady-state amperometric current responses of MX-GCE towards H_2_O_2_ (25, 50, and 100 µM) in the presence of interfering species such as Glc, UA, AA, DA, and Cl^−^ (5 µM) in 0.1 M PBS (pH = 7.4).

The selectivity of the MX-GCE towards H_2_O_2_ was evaluated by amperometric experiments at a potential of −0.45 V in 0.1 M PBS (pH 7.4) in the presence of common electroactive interferents, including glucose (Glc), uric acid (UA), ascorbic acid (AA), dopamine (DA), and chloride ions (Cl^−^). As illustrated in Fig. 8a, successive additions of 5 µM Glc, UA, AA, DA, and sodium chloride resulted in negligible changes in the cathodic current, indicating minimal interference with its H_2_O_2_ sensing ability. In contrast, the stepwise standard additions of H_2_O_2_ (2.5, 5, and 10 µM) produced a well-defined increase in current, demonstrating high selectivity of the MX-modified electrode towards H_2_O_2_. Fig. 8b depicts the effect of interfering species response with respect to H_2_O_2_ plotted as a bar diagram. This showed that at a suitable negative potential, H_2_O_2_ undergoes efficient electrocatalytic reduction at Mo-active sites, while the oxidation or reduction of these common interferents is kinetically suppressed. This behaviour confirmed that the obtained results validated the MX-GCE for reliable detection of H_2_O_2_ in complex biological environments.^87^

## 6. Real-world sample and cellular analysis

### 6.1. Direct spiking in the cell medium

To analyse the activity of MX-GCE in biological environments, a known concentration of cell suspension (2 × 10^−6^ cells per mL) was diluted in 0.1 M PBS (pH 7.4) immediately before the experiment to maintain the viability of the cells at room temperature. A known concentration of H_2_O_2_ (0.5 mM) was then added to the cell medium, and the amperometric response was recorded with continuous stirring at 720 rpm. Another experiment was carried out where separate additions of the same H_2_O_2_ concentration were made into PBS, without the cells, and used as a control. These results, repeated thrice, were then used to calculate the recovery and RSD of the H_2_O_2_ from the spiked samples. The estimated values are depicted in Table 3. The difference in the concentrations of H_2_O_2_ added and found is probably due to the positive matrix effect that usually occurs in biological environments due to the presence of various endogenous substances.^88^ This is neglected as the estimated RSD is between 1% and 2.5%, which indicated excellent reproducibility. The obtained recovery% is higher than 100% which denoted the matrix effects of a biological sample. As the cell concentration is diluted, the interference from the biological sample is found to be minimal.^89^

**Table 3:** Real-world sample analysis in PBS containing SW480 cells

S. no.	Added (mM)	Found (mM)	Recovery%	RSD%
1	0.5	0.67	118.6	2.2
2	1	1.19	109.2	1.2
3	1.5	1.68	107.0	1.0

### 6.2. Extracellular generation of H_2_O_2_

**Fig. 9:**
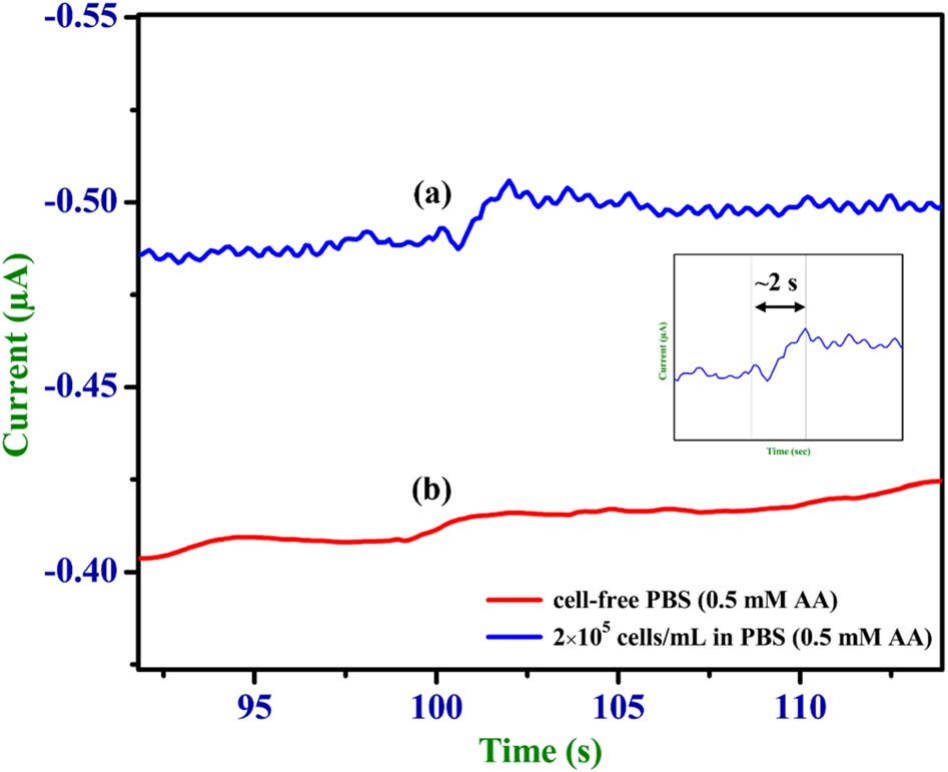
(a) Amperogram recorded in the presence of 0.5 mM AA at −0.45 V in 0.1 M PBS (pH 7.4) (stirring rate = 720 rpm) in PBS containing 2 × 10^5^ cells per mL. (b) Amperogram recorded in the presence of 0.5 mM AA at −0.45 V in 0.1 M PBS (pH 7.4) (stirring rate = 720 rpm) without cells. Inset: magnified view of the brief current response with a response time of ∼2 s.

Real-time monitoring of H_2_O_2_ released by cells was carried out upon successive additions of AA (0.5 mM) to a cell-containing PBS (2 × 10^5^ cells per mL) under constant stirring (720 rpm). All measurements were performed in 0.1 M PBS (pH of 7.4) at an applied potential of −0.45 V, which selectively favours the reduction of H_2_O_2_ while suppressing any direct electrochemical responses from AA. As depicted in Fig. 9, each addition of AA in the presence of cells produced a distinct increase in cathodic current (Fig. 9a), indicating extracellular H_2_O_2_ triggered by AA stimulation.^85^ On the other hand, the same conditions were carried out in a cell-free PBS. It shows no significant increase in current upon AA addition, confirming that AA did not interfere with the amperometric response (Fig. 9b). Minor fluctuations observed in the blank are attributed to the possible disturbances during solution addition rather than the electrochemical process. The current responses obtained from three independent experiments were then used to plot the calibration curve. It reveals the linear relationship between the concentration and the current response with *R*^2^ of 0.999, highlighting the excellent reproducibility of the sensor. Furthermore, the current response time after the addition of the stimulant was ∼2 s (Fig. 9, inset), which reinforces the ability of AA to generate ROS extracellularly. The concentration of the H_2_O_2_ generated extracellularly was calculated and given in Table 4,^90^ which also revealed that the conversion efficiency increases with AA concentration.

**Table 4:** Ascorbic acid-induced H_2_O_2_ generation and corresponding conversion efficiency

S. no.	AA concentration added (mM)	H_2_O_2_ concentration found (mM)	Conversion efficiency (%)
1	0.5	0.1	20
2	1.0	0.6	60
3	1.5	1.14	70

The concentration of AA (0.5–1.0 mM) used for stimulation exhibits pro-oxidant activity in the cancer cells, causing enhanced release of ROS. In extracellular environments, AA undergoes oxidation to form an ascorbate radical, which further initiates H_2_O_2_ generation, which is detected by amperometry experiments.^91^ Previously reported studies established IC_50_ values of 1.64 mM, when ascorbate was used for 3 hours. This denoted that the cell viability at these concentrations decreases only by 40–60%, causing cell death after 24 hours.^36,90^

## 7. Cell viability assay

The biocompatibility of the ascorbic acid was assessed using the MTT assay in the SW480 cell line. Cells were seeded in 96-well plates at a density of 7 × 10^3^ cells per well and incubated for 24 h at 37 °C in a humidified 5% CO_2_ atmosphere to allow cell attachment. Following incubation, cells were treated with ascorbic acid (0.5 mM) and incubated for 24 h. Untreated cells were maintained as the negative control. After treatment, the culture medium was carefully removed, and 100 µL of MTT solution (prepared from a 5 mg mL^−1^ stock to obtain a final concentration of 0.5 mg mL^−1^) was added to each well. The plates were incubated for 3 h at 37 °C to facilitate the formation of formazan crystals. Subsequently, the MTT solution was discarded, and the resulting formazan crystals were solubilized by adding 100 µL of DMSO per well, followed by incubation at 37 °C for 10 min. Absorbance was measured at 570 nm using a microplate reader. At 3 h of AA treatment, up to 81.9% of the cells remained viable. At 6 h, 64% of the cells remained viable. Fig. S8 shows the phase-contrast microscopic images of SW480 cells under 0.5 mM AA stimulation at 3 h and 6 h with untreated controls. Therefore, it is evident that AA stimulation successfully induced oxidative stress without killing living cells. All experiments were performed in triplicate. Cell viability was determined using eqn (14).14
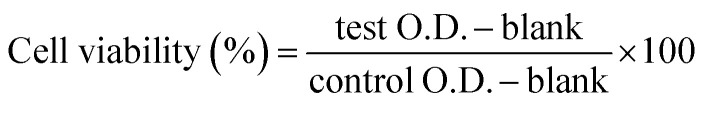


## 8. Conclusion

In this work, a non-enzymatic electrochemical sensing platform based on Mo_2_CT_*x*_ MXene was successfully developed for the selective and sensitive detection of H_2_O_2_, with its application in cancer cell environments. Various structural and functional characterisation methods, such as UV-vis, FTIR, XRD, SEM, EDS, and elemental mapping, were employed, which confirmed the successful synthesis of Mo_2_CT_*x*_ MXene. The material exhibited distinct electrocatalytic activity towards H_2_O_2_ reduction at a low operating potential of −0.45 V. The as-prepared sensor demonstrated a linear response from 0.49 to 78.3 µM, with an excellent correlation coefficient of *R*^2^ = 0.9956. A low LOD of 1.02 µM was achieved, highlighting the capability of the MXene interface to detect biologically relevant concentrations of H_2_O_2_. The rapid response time (∼2 s), good repeatability, and minimal interference from common electroactive species further reinforced the sensitivity and selectivity of the platform. The practical applicability of the sensor was confirmed through real sample analysis and by cellular studies, which demonstrated reliable quantification in cell-containing media. Real-time measurements of extracellular H_2_O_2_ generated after ascorbic acid stimulation were also provided. Overall, this work established Mo_2_CT_*x*_ MXene as a stable and effective material to develop H_2_O_2_ sensors which can be used to develop point-of-care detection tools for applications like oxidative stress monitoring and cancer diagnostics.

## Author contributions

S. V.: writing the original draft, formal analysis, methodology, data curation, and conceptualization. V. M.: methodology, data curation. C. G.: writing, editing and review. R. A.: writing, review and editing. D. G.: review and editing. S. A.: writing, review and editing. A. K. S.: writing, editing, supervision, resources, visualization, validation, project administration, and funding acquisition.

## Conflicts of interest

The authors declare that they have no known competing financial interests.

## Supplementary Material

NA-008-D6NA00025H-s001

## Data Availability

All data generated and analysed during this study are available within the manuscript and its supplementary information (SI) file. Supplementary information: XRD pattern of the Mo_2_Ga_2_C MAX phase precursor, FESEM images of the MAX phase, scan rate-dependent cyclic voltammograms, inter- and intra-day stability data, pH optimization studies, catalyst loading optimization, and phase-contrast microscopy images of SW480 cells. See DOI: https://doi.org/10.1039/d6na00025h.
