# Supplementary material for: Mo2CTx MXene-based non-enzymatic electrochemical sensor for selective detection of hydrogen peroxide in colorectal cancer cells
Source: Nanoscale Adv. 2026 May 19;8(14):3993–4007. doi: 10.1039/d6na00025h (PMC13245807; doi:10.1039/d6na00025h)
Supplement: NA-008-D6NA00025H-s001 [file NA-008-D6NA00025H-s001.pdf]

Supplementary Information (SI) for Nanoscale Advances.
This journal is © The Royal Society of Chemistry 2026

Supporting Information

Mo_2CT_x MXene–Based Non-Enzymatic Electrochemical Sensor for Selective Detection of Hydrogen Peroxide in Colon Cancer Cells

Shruthi Venkataraman[1,#], Vasanth Magesh[1], Chandramohan Govindasamy[2], Raji Atchudan[3,#], Sandeep Arya[4], and Ashok K. Sundramoorthy[*,1]

[1]Centre for Nano-Biosensors, Department of Prosthodontics and Materials Science, Saveetha Dental College and Hospitals, Saveetha Institute of Medical and Technical Sciences, India.

[2]Department of Community Health Sciences, College of Applied Medical Sciences, King Saud University, P.O. Box 10219, Riyadh 11433, Saudi Arabia.

[3]School of Chemical Engineering, Yeungnam University, Gyeongsan 38541, Republic of Korea.

[4]Department of Physics, University of Jammu, Jammu 180006, Jammu and Kashmir, India.

* Corresponding author.

E-mail addresses: ashok.sundramoorthy@gmail.com (A.K. Sundramoorthy).

[#]These authors equally contributed.

List of Supplementary Figures

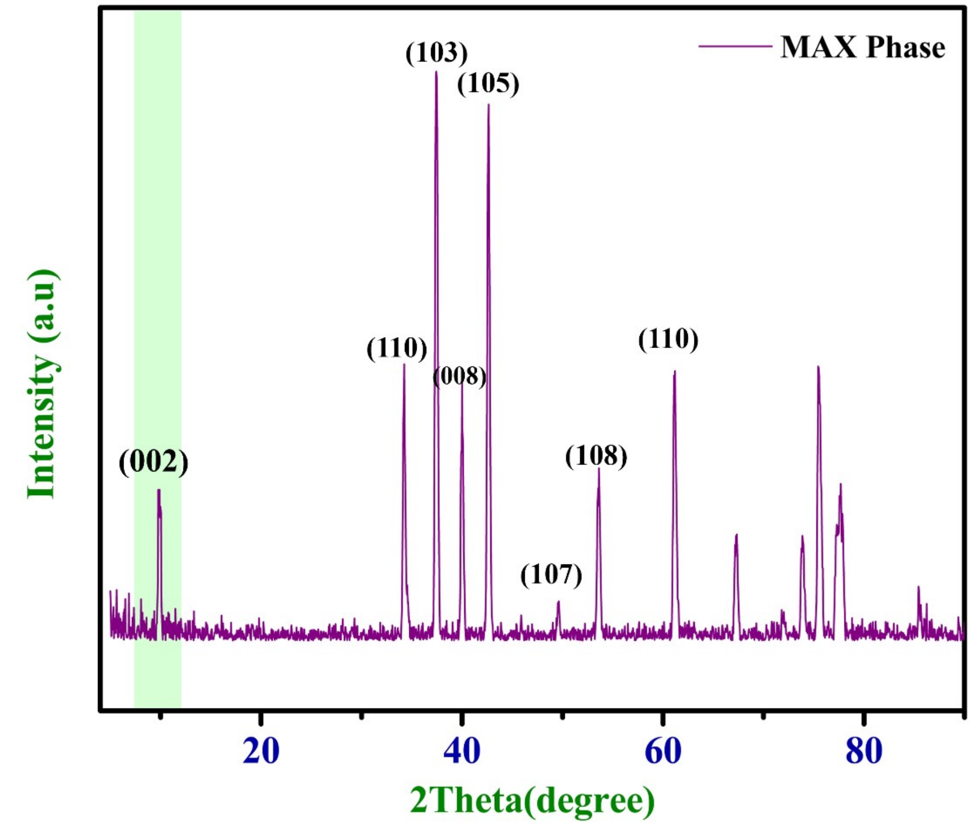

Figure S1. XRD data of Mo_2Ga_2C MAX phase.

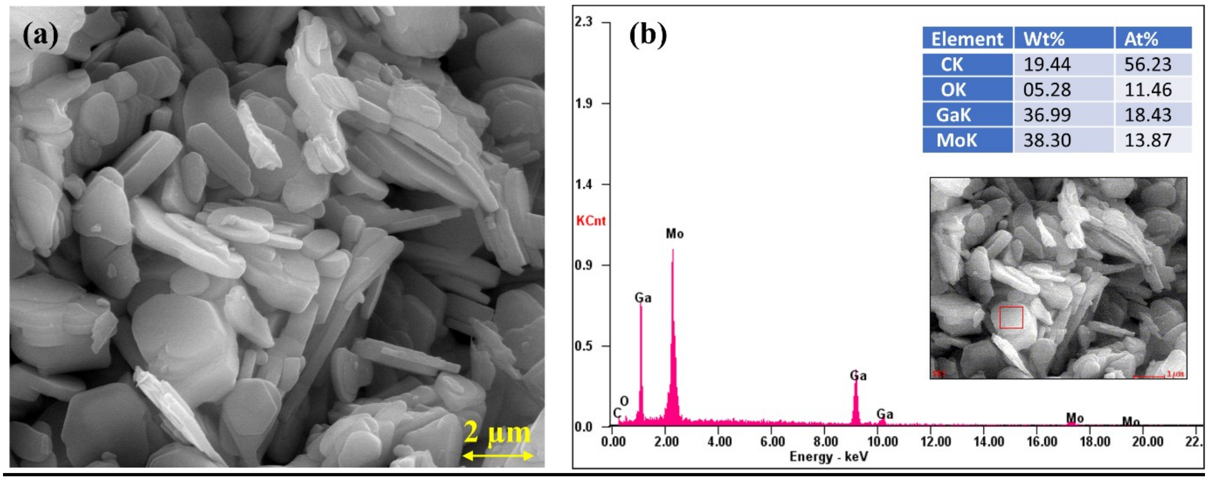

Figure S2. (a) SEM micrographs of Mo_2Ga_2C MAX phase precursor (Scale bar - 2 µm), (b) EDAX spectrum of Mo_2Ga_2C MAX phase. Inset: Table shows the list of elements and their wt% and at% along with SEM micrograph of Mo_2Ga_2C.

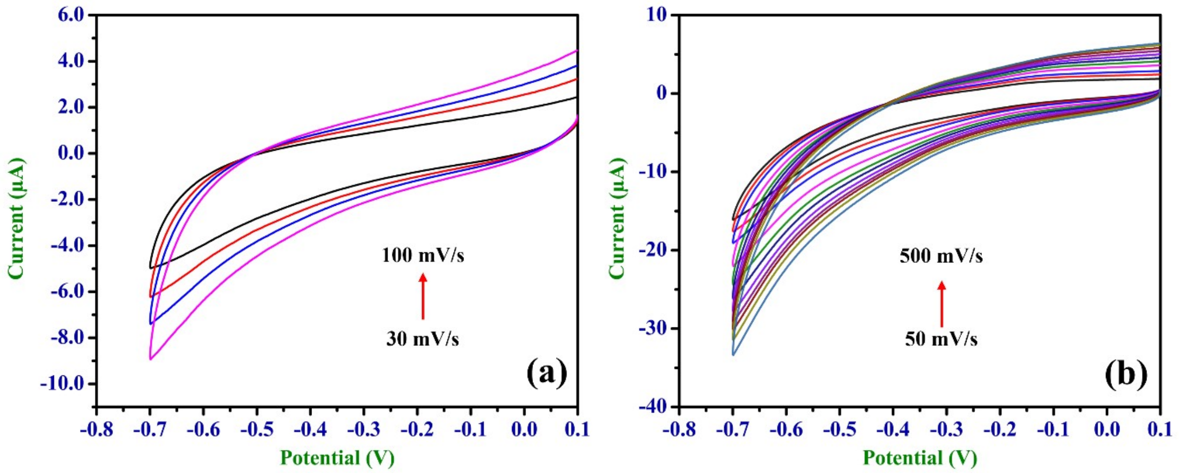

Figure S3. (a) CVs were recorded at different scan rates using an MX-GCE (without H_2O_2) in 0.1 M PBS (pH=7.4) from 30 mV/s to 100 mV/s. (b) CVs were recorded at different scan rates from 50 mV/s to 500 mV/s using an MX-GCE in 0.1 M PBS (pH=7.4) containing 284 mM H_2O_2.

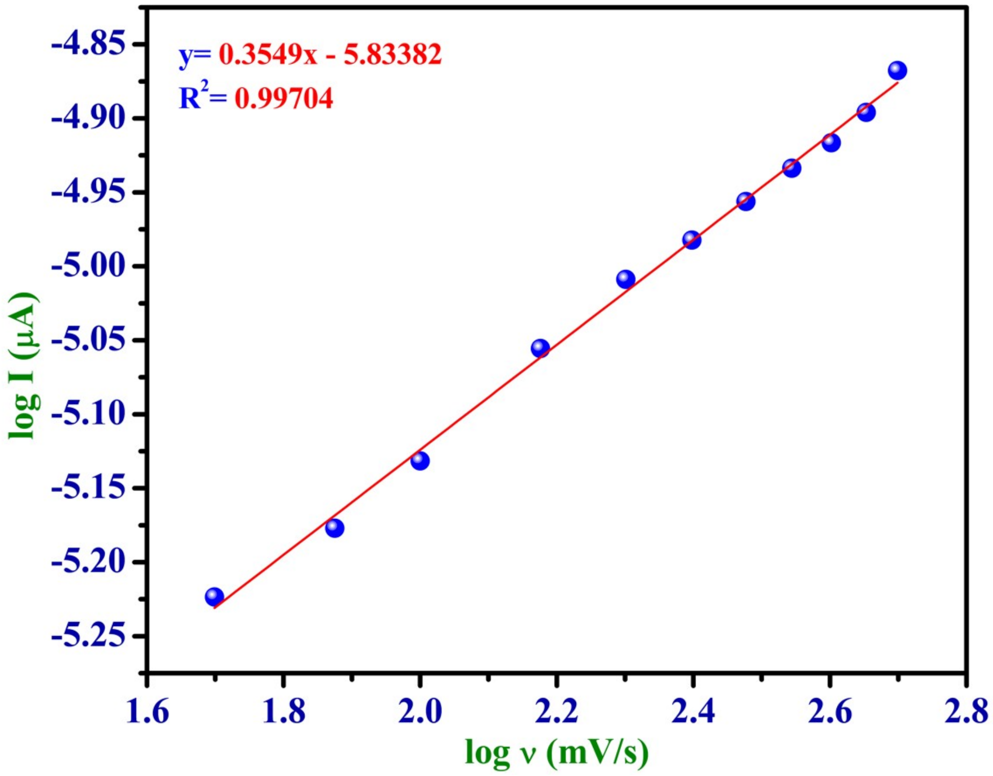

Figure S4. Linear plot of log I (μA) vs log ν (mV/s) with different scan rates at a fixed potential of -0.45 V.

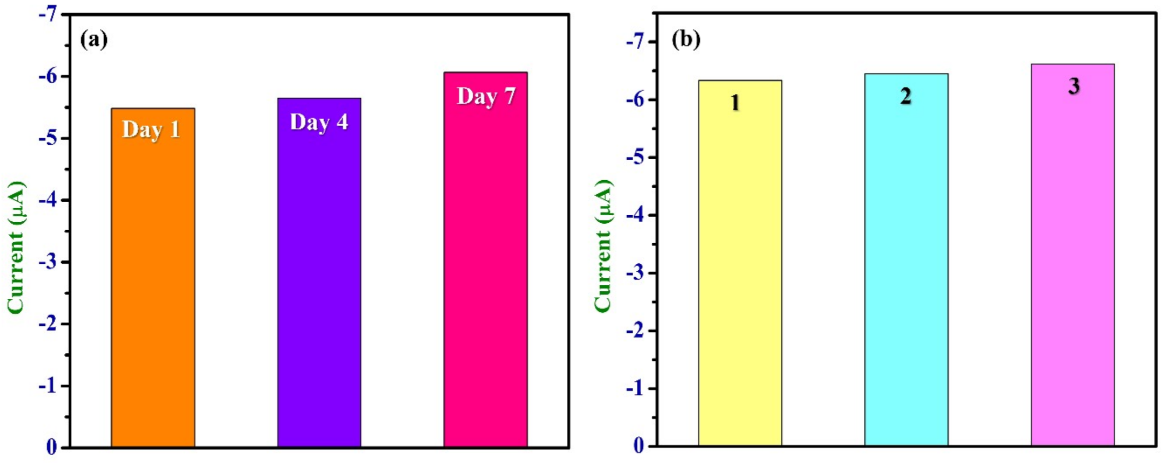

Figure S5. (a) CVs were recorded using MX-GCE in 0.1 M PBS (pH=7.4) at a scan rate of 50 mV/s on day 1, day 4, and day 7. (b) CVs were recorded using MX-GCE in 0.1 M PBS at a scan rate of 50 mV/s (n=3) for intra-day stability measurements.

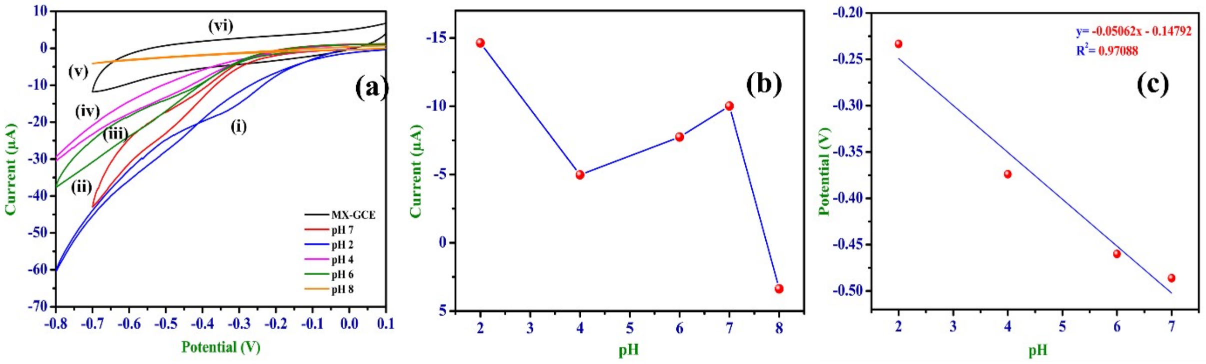

Figure S6. (a) CVs were recorded using a MX-GCE in the presence of 284 mM H_2O_2 with various buffer solutions at different: pH = (i) 2, (ii) 7.4, (iii) 6, (iv) 4, (v) 8, in comparison with CVs recorded with MX-GCE in the absence of H_2O_2 in 0.1 M PBS (pH = 7.4) at a scan rate of 50 mV/s. (b) Cathodic current response vs different pH in the presence of 284 mM H_2O_2. (c) A linear plot shows the corresponding H_2O_2 reduction potential vs different pH in the presence of 284 mM H_2O_2.

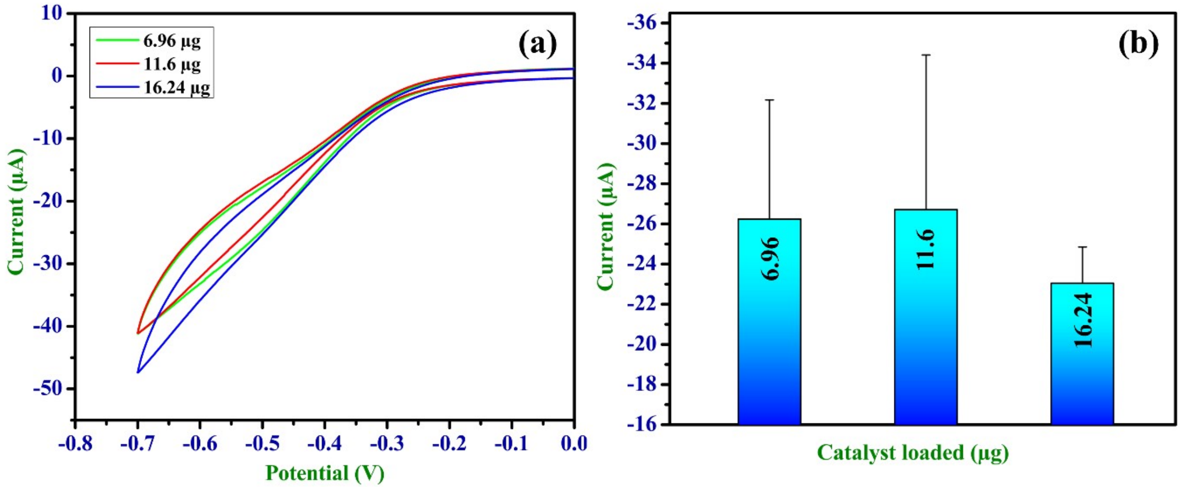

Figure S7. (a) CVs were recorded using an MX-GCE modified with different amount of catalyst (6.96, 11.6, 16.24 μg) in 0.1 M PBS (pH 7.4) in the presence of 284 mM H_2O_2 at a scan rate of 50 mV/s. (b) Corresponding bar plot of cathodic current vs different catalyst concentrations in 0.1 M PBS (pH = 7.4) in the presence of 284 mM H_2O_2 with standard deviation (SD) of 5.94×10^{-6}, 7.70×10^{-6}, 1.81×10^{-6} for 6.6 μg, 11.6 μg, and 16.2 μg, respectively.

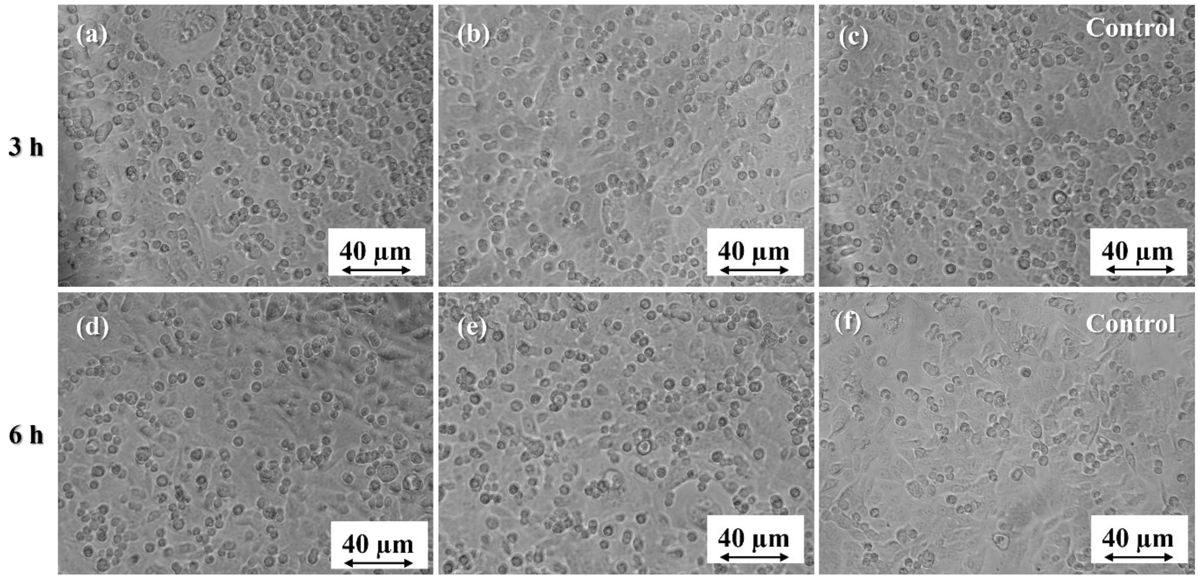

Figure S8. Phase contrast optical microscopy images of SW480 colorectal cancer cells following treatment with 0.5 mM ascorbic acid for 3 h at 37 °C, (a,b) different fields of view

from AA-treated SW480 cells for 3 h, (c) untreated controls (SW480 cells) without 0.5 mM AA for 3 h at 37 °C, (d, e) different fields of view from AA-treated SW480 cells for 6 h, and (e) untreated controls without 0.5 mM AA for 6 h at 37 °C.
